# A rigorous assessment and comparison of enumeration methods for environmental viruses

**DOI:** 10.1038/s41598-020-75490-y

**Published:** 2020-10-29

**Authors:** Judith Kaletta, Carolin Pickl, Christian Griebler, Andreas Klingl, Rainer Kurmayer, Li Deng

**Affiliations:** 1 Institute of Virology, Helmholtz Centre Munich, Munich, Germany; 2 Institute of Virology, Technical University Munich, Munich, Germany; 3 Institute of Groundwater Ecology, Helmholtz Centre Munich, Munich, Germany; 4 Department for Limnology and Bio-Oceanography, University of Vienna, Vienna, Austria; 5 Plant Development and Electron Microscopy, Department Biology I, Biocenter, Ludwig-Maximilians-University Munich, Munich, Germany; 6 Research Department for Limnology, University of Innsbruck, Innsbruck, Austria

**Keywords:** Microbiology techniques, Microbial ecology

## Abstract

Determining exact viral titers in a given sample is essential for many environmental and clinical applications, e.g., for studying viral ecology or application of bacteriophages for food safety. However, virus quantification is not a simple task, especially for complex environmental samples. While clonal viral isolates can be quantified with relative high accuracy using virus-specific methods, i.e., plaque assay or quantitative real-time PCR, these methods are not valid for complex and diverse environmental samples. Moreover, it is not yet known how precisely laser-based methods, i.e., epifluorescence microscopy, flow cytometry, and nanoparticle tracking analysis, quantify environmental viruses. In the present study, we compared five state-of-the-art viral quantification methods by enumerating four model viral isolates of different genome and size characteristics as well as four different environmental water samples. Although Nanoparticle tracking analysis combined with gentle staining at 30 °C could be confirmed by this study to be a reliable quantification technique for tested environmental samples, environmental samples still lack an universally applicable and accurate quantification method. Special attention has to be put on optimal sample concentrations as well as optimized sample preparations, which are specific for each method. As our results show the inefficiency when enumerating small, or single-stranded DNA or RNA viruses, the global population of viruses is presumably higher than expected.

## Introduction

Numbers of viral particles in the environment vary greatly, ranging from 10^7^ to 10^9^ particles g^–1^ dry weight in soil and 10^9^ to 10^10^ particles L^–1^ in water, but can even be as high as 10^12^ particles L^−1^ in hypersaline environments, while approximately 10^5^ viruses per m^3^ have been reported in the air^1–6^. Generally, the more oligotrophic the environment is, the fewer viral particles are present^7–9^. Based on these values, the total virus abundance in the global marine system has been projected to be as high as 10^30^ viral particles. Thus, viral particles are considered to be the most abundant biological entities on Earth^10^. However, the quantification of nano-sized viral particles in a sample of unknown composition is an essential but sophisticated task that remains challenging. For fundamental parameters in studies of viral ecology or general or medical virology, such as virus-to-bacteria ratio, an accurate estimation of viral particles is essential.

Various quantification techniques for small biological particles are available based on different biological or physical theories. They can be classified into two groups according to the available details of the respective virus. When viral genetic information is existing or the viral host can be cultivated, targeted quantification such as quantitative real-time polymerase chain reaction (qPCR), plaque-based assay (PA), or most probable number assay (MPN) can be performed. A rather recent advancement of the polony method to quantify diverse viruses that belong to distinct viral families has been made by Lindell et al*.*^11^. This method circumvents biases in the amplification of diverse templates by preventing the competition between templates through the physical separation of template molecules, which would be for example the case when degenerated primers are used in qPCR assays. Is genetic information lacking, viral particles can still be quantified using epifluorescence microscopy (EPI), transmission electron microscopy (TEM), flow cytometry (FCM), or nanoparticle tracking analysis (NTA).

The first method, qPCR, is a molecular detection technique that requires knowledge of conserved viral genome sequences. Virus-specific primers allow the amplification and quantification of a specific viral gene sequence. As viral genomes are generally very unique and universal primers are lacking (e.g., 16S rRNA-targeting primers for bacteria), only viruses with (at least partly) known genomes can be quantified with qPCR. With this method, the presence or absence of a given gene sequence can be detected, but further information relating to the infectiousness or particle integrity of the virus are beyond its capacity. Nonetheless, this method is seen as the gold standard of viral quantification techniques. In contrast, PA and MPN are based on the identity of the host that is susceptible to a certain virus. For PA, host cells and the virus-containing samples are incubated in a solidifying medium. The virus can then be detected by the formation of clear spots in a turbid lawn formed by the host cells. Consequently, the integrity and infectiousness of a virus particle is a requirement for PA. With MPN, different dilutions of a viral sample are co-incubated with liquid host culture, and the presence of the virus is determined based on lysis of the host cells, measured spectrophotometrically on successive days^12^. Here as well, infectiousness of viral particles is a prerequisite.

Methods that quantify independently of additional information about the virus or its host identity struggle with the small sizes of viral particles. Thus, all of these methods require a staining procedure to distinguish viral from non-biological particles. For EPI, virus-sized particles are captured on a fine-mesh filter and subsequently stained with a fluorescent nucleic acid dye (e.g., SYBR green, SYBR gold, or Yo-Pro1)^13^. Particles are generally visualized under an epifluorescence microscope at 1000 × magnification. In addition, FCM, initially developed for the identification, quantification, and separation of eukaryotic and prokaryotic cells has been adapted as a high-throughput method for counting viral particles^14,15^. Again, in order to distinguish viral particles from background noise, viral nucleic acid is fluorescently labelled. Particles are singularized in a liquid stream, passing a laser beam that is suitable for the dye, and thus being quantified according to their fluorescence and scatter signal. A fairly new approach to determine viral concentrations is NTA using the NanoSight Instrument (Malvern Pananalytical Ltd., Malvern, United Kingdom). A microscope visualizes the Brownian motion of small particles in liquid phase in real-time and relates it to their particle size. Additional lasers allow the assessment of fluorescence signals to specifically track particles^16–19^.

All of the methods presented above have their advantages and drawbacks as well as specific fields of application (Table 1). However, the accuracy of the different methods is arguable, especially of those methods that aim to quantify uncharacterized, composited samples. The aim of the present study is to compare different viral quantification techniques in terms of an accurate and precise enumeration of both, pure viral strains and environmental water sample of unknown viral composition. We cross-compared the accuracy of measurement of five different viral quantification techniques: (i) PA, (ii) qPCR, (iii) EPI, (iv) FCM, and (v) NTA. Firstly, four clonal *Escherichia coli* bacteriophage isolates (T4, T7, ϕX174, and MS2) with different structural properties (Table 2) were quantified using each method. Different staining procedures were followed where applicable: Staining at 80 °C for 10 min is the standard procedure for FCM sample preparations^14^. However, others recommended also lower staining temperatures^20^. We included therefore another staining procedure conducted under gentler conditions at 30 °C but with a prolonged incubation time. By comparing all measurements against the qPCR results, as here DNA targets are quantified rather accurately^21^, the optimal preparation procedure and method for an exact and reliable quantification of viral particles shall be identified. This knowledge can then be transferred and verified on four aquatic samples (ground, lake, river and wastewater). These samples contained mixed viral communities of unknown compositions; consequently, specific techniques such as PA or qPCR could not be applied.

**Table 1:** Specifications, advantages, and disadvantages of the virus quantification techniques presented.

	Plaque assay	Epifluorescence microscopy	Flow cytometry	Nanoparticle tracking analysis	Quantitative real–time PCR
Size limitation	N/A	> 200 nm	0.5–40 µm	10–2000 nm	N/A
Measurable features	Active/lytic viral particles	Total viral particles	Total viral particles	Total viral particles	Specific gene abundance
Optimal concentration	40–400 PFU mL^−1^	10^7^–10^8^ VLP mL^−1^	10^6^–10^7^ VLP mL^−1^	10^7^–10^9^ VLP mL^−1^	10^6^–10^8^ VLP mL^−1^
Time requirement per sample	Minimum of 24 h	Around 2 h	Around 30 min	Around 15 min	Around 4 h
Advantages	Measures infective particles, low consumable costs	Low operating and consumable costs, fast results, visible control	Low to high operating and consumable costs, fast results	Low operating and consumable costs, direct particle visualization	Good reproducibility, accurate quantification of specific genes
Disadvantages	Many dilution steps needed, poor reproducibility, result not immediately available, cultivable host needed	High initial costs for equipment, DNA-bound non-viral particles may be counted as well	High initial costs for equipment, DNA-bound non-viral particles may be counted as well	High initial costs for equipment	Phage-specific primers needed, DNA extraction needed (no direct quantification possible), high costs

**Table 2:** Characteristics of *Escherichia coli* bacteriophage isolates used in the present study.

	T4	T7	MS2	ϕX174
Family	*Myoviridae*	*Podoviridae*	*Leviviridae*	*Microviridae*
DSM ID	4505	4623	13,767	4497
Tail length	200 nm	28.5 nm	No tail	No tail
Capsid diameter	57 nm	55 nm	27 nm	30 nm
Genome type	dsDNA	dsDNA	ssRNA	ssDNA
Genome size	160 kbp	40 kbp	4 kb	5 kb
Bacterial host	*E. coli* (DSM 613)	*E. coli* (DSM 613)	*E. coli* (DSM 5695)	*E. coli* (DSM 13127)
Citation	^22^	^22^	^22^	^23^

## Materials and methods

### Bacteriophages

Four lytic *E. coli*-specific phages were used in the present study: MS2 (DSM 13767), T4 (DSM 4505), T7 (DSM 4623), and ϕX174 (DSM 4497). The genomic and structural properties of the phages as well as their bacterial hosts are listed in Table 2. For preparation of the virus isolate stocks, the respective bacterial host was grown in sterile LB medium (LB broth Miller, Sigma-Aldrich, St. Louis, Missouri) until an optical density of 0.3 measured at 600 nm was reached, then inoculated with phages at a virus-to-bacteria-ratio of 0.1, followed by overnight incubation. Remaining bacterial cells were killed by the addition of 1/10 volume of chloroform for 1 h. After separation from the bacterial cell debris, virus stocks were filtered with 0.22 µm syringe filters (Millex-GP, Merck-Millipore, Billerica, Massachusetts) and filtration was repeated prior preparation of samples for measurements.

### Environmental samples

Environmental samples were collected from four different aquatic habitats: the income water tank of a wastewater treatment plant (Gut Großlappen, Munich, Germany), an on-site groundwater collection well (48°13′25.8" N 11°35′45.4" E, Munich, Germany), a lake (Feldmochinger See; 48°12′56.0" N 11°30′49.4" E, Munich, Germany), and a river (Isar; 48°32′59.3" N, 12°10′42.4" E, Landshut, Germany). To remove particles the size of bacteria and larger, all water samples were filtered with 0.22 µm syringe filters (Millex-GP). Measurements with flow cytometer and nanoparticle tracking analysis were performed simultaneously and on the sampling day. Quantification with epifluorescence microscopy as well as DNA extraction was conducted on the next day. Samples were stored in 4 °C.

Additionally, a mixed water sample (lake and wastewater) with an approximate concentration of 10^8^ virus-like particles per mL (VLP mL^–1^) was prepared. This sample was spiked with 1× 10^8^, 5× 10^8^ and 1× 10^9^ T4 particles mL^−1^. Before the addition, phage T4 stock has been quantified with qPCR.

### Viral quantification

All measurements were performed in biological and technical duplicates.

### Plaque assay (PA)

The PA was performed using a soft agar overlay technique as described elsewhere^24^. Briefly, 0.5 mL of appropriate dilutions of phages were mixed with an equal volume of fresh cultures of the corresponding hosts, grown overnight (incubated in LB medium at 37 °C until an optical density of 0.3 measured at 600 nm was reached). The phage-bacteria-suspension was mixed with 3 mL warm soft agar (0.75% w/v agar and 2.5% w/v LB) and gently poured on a petri dish already containing an LB agar layer (1.5% w/v agar and 2.5% w/v LB) in biological and technical replicates. Upon solidification, the petri dishes were incubated bottom up for overnight at 37 °C. After 15–20 h, depending on the bacterial growth efficiency, the plaques formed were manually counted and the phage titers as plaque-forming units per mL (PFU mL^–1^) were calculated.

### Flow cytometry (FCM)

All samples were prepared as described previously with some adaptations^14^. We decided on these modifications based on the publications of Tomaru and Nagasaki (2007) and Brum and colleagues (2013). More precisely, samples were not fixed with glutaraldehyde after sampling as this may decrease the fluorescence intensity as well as the viral counts. Tomaru and Nagasaki concluded, that a fixation does not necessarily improve the staining ability of the virus particles^20^. Besides, our samples were measured immediately on the day of sampling, thus a preservation of the viral particles was not necessary. Another step recommended by Brussaard (2004) we did not follow is the flash freezing of the viral sample in liquid nitrogen. It has been shown that nitrogen fixation hampers the preparation procedure for TEM resulting *inter alia* in morphology changes^25^. To what extent particles would be enumerated correctly after fixation and nitrogen treatment with nanoparticle tracking analysis where particle integrity would certainly play a role during the enumeration process, is also debatable. As consequence, we decided, to omit this step in order to maintain a consistent sample handling and accomplish comparable conditions for all methods.

In brief, samples were diluted appropriately with sterile, filtered PBS buffer (0.02 µm Anotop 25 syringe filter, Whatman, Maidstone, UK; Sigma Aldrich) to fulfill the instrument’s optimal concentration requirements of approximately 10^6^ VLP mL^–1^ (Table 1). Fluorescent TRUCOUNT beads (BD, Becton, Dickinson and Company, Franklin Lakes, New Jersey) were added to each sample as an internal reference. The samples were stained with 1 × SYBR gold nucleic acid stain (Thermo Fisher, Waltham, Massachusetts) and incubated either for 10 min at 80 °C (FCM80) or for 1 h at 30 °C (FCM30) prior to measurement. Tomaru & Nagasaki recommended an incubation at room temperature, as higher temperatures reduced the viral counts. We chose therefore two staining temperatures, one at 80 °C, following the suggestion of Brussaard^14^ and one at 30 °C, following the reference of Tomaru & Nagasaki^20^.

All samples were measured with a FC500 flow cytometer equipped with an air-cooled 488 nm Argon ion laser (Beckman Coulter, Brea, California) in biological and technical replicates. Analysis and evaluation of the samples was performed using StemCXP Cytometer software (v2.2).

### Nanoparticle tracking analysis (NTA)

Viral isolate samples were diluted appropriately with sterile phage buffer (10 mM Tris [pH 7.5], 10 mM MgSO_4_, and 0.4% w/v NaCl) to obtain the optimal concentration range of 10^7^–10^9^ VLP mL^–1^ (Table 1). Afterwards, samples were either untreated or stained with 1 × SYBR gold for 10 min at 80 °C or 1 h at 30 °C (NTA80 or NTA30, respectively). Each sample was injected manually into the machine’s specimen chamber with a sterile 1 mL syringe (Braun, Melsungen, Germany), and measured three times for 20 sec at room temperature in three independent preparations. Samples were measured using a NanoSight NS300 (Malvern Pananalytical Ltd., Malvern, United Kingdom) equipped with a B488 nm laser module and a sCMOS camera, following the manufacturer’s protocol. Analysis was performed with the NTA 3.1 Analytical software (release version build 3.1.45).

### Epifluorescence microscopy (EPI)

Staining of the samples was carried out as described by Patel et al.^26^. Briefly, all samples were diluted appropriately with 0.02 µm filtered 1 × TE buffer (pH 7.5, AppliChem, Darmstadt, Germany) to a concentration of 10^7^ particles mL^–1^. For environmental samples with lower concentrations, a volume of 10 mL was used.

Then, 1 mL of each diluted sample (10 mL of environmental samples) was passed through a 0.02 µm Anodisc filter (Whatman) in duplicates. After complete desiccation, the filter was stained using a drop of 2 × SYBR gold dye (Thermo Fisher) with the virus side up, and incubated at room temperature for 15 min in the dark. Stained filters were mounted on a glass slide with 20 µL antifade solution (Thermo Fisher). Slides were analyzed using an Axiolab fluorescence microscope (Carl Zeiss, Oberkochen, Germany) equipped with a 488 nm laser. A camera was used to take ten pictures per sample, which were analyzed using ImageJ (version 1.50i). Numbers of particles on the whole filter were calculated by multiplying the counts with the quotient of the area of the filter by area of the pictures.

### Quantitative real-time PCR (qRT-PCR)

Prior to the DNA extraction 1 mL of sample has been treated with DNase as described previously with a modified incubation procedure for one hour at 37 °C^27^. The DNA extraction has been conducted from the complete volume after DNase treatment using the Wizard® PCR Preps DNA Purification Resin and Minicolumns (Promega, Madison, Wisconsin) as previously described^28^. RNA was extracted with a QIAmp MinElute Virus Spin Kit (total volume of 1 mL sample) (Qiagen, Hilden, Germany) and cDNA was synthesized using a DyNAmo cDNA Synthesis Kit (Thermo Fisher) according to the manufacturers protocols. For all samples, DNA or RNA was isolated in duplicates.

T4 was quantified using primers amplifying a 163 bp region of the gp18 tail protein (T4F 5′-AAGCGAAAGAAGTCGGTGAA-3′ and T4R 5′-CGCTGTCATAGCAGCTTCAG-3′)^29^. For T7, primers amplifying a 555 bp segment of gene 1 (T7_4453F 5′-CTGTGTCAATGTTCAACCCG-3′ and T7_5008R 5 ‘-GTGCCCAGCTTGACTTTCTC-3′)^30^. ϕX174 was quantified using primers specific for the capsid protein F (ϕX174F 5′-ACAAAGTTTGGATTGCTACTGACC-3′ and ϕX174R 5′-CGGCAGCAATAAACTCAACAGG-3′) resulting in a 122 bp fragment^31^. For MS2, primers amplifying a 314-bp fragment (MS2_2717F 5′-CTGGGCAATAGTCAAA-3′ and MS2_3031R 5′-CGTGGATCTGACATAC-3′) were used^32^. Quantitative PCR was performed in a total volume of 20 µL consisting of 10 µL Brilliant III Ultra-Fast QPCR Master Mix (Agilent, Santa Clara, California), 5 µL DNA template or PCR-grade water as a negative control, as well as the following optimized primer concentrations (supporting information): 0.5 µM primers T4F and T4R, 0.8 µM primers T7_4453F and T7_5008R, 0.6 µM primers ϕX174F and ϕX174R, or 0.3 µM primers MS2_2717F and MS2_3031R, respectively. The amplifications were run on a Mx3000P qPCR system (FAM/SYBR® Green I filter [492 nm–516 nm], OS v7.10, Stratagene, San Diego, California) with the following cycling conditions: T4: 95 °C for 10 min, (95 °C for 15 sec, 60 °C for 1 min, 72 °C for 1 min) for a total of 45 cycles, T7: 95 °C for 12 min, (95 °C for 30 sec, 58 °C for 30 sec, 72 °C for 1 min) for a total of 30 cycles, ϕX174: 94 °C for 3 min, (94 °C for 15 sec, 60 °C for 1 min) for a total of 40 cycles, and MS2: 95 °C for 10 min, (95 °C for 15 sec, 50 °C for 30 sec, 72 °C for 30 sec) for a total of 45 cycles. Each replicate was measured four times. Analysis of the melting curves confirmed the specificity of the chosen primer as no variations compared to the standard melting curves could be observed. Standard curves were prepared using the appropriate dilutions of gblocks gene fragments (IDT, Coralville, Iowa) of the respective viral DNA in PCR-grade water (supporting information, Tables S1 and S2). Data analysis was performed using the manufacturer’s MxPro Mx3000P software (v4.10).

### TEM preparation

Although TEM may be used for quantification, only the virus morphology and integrity upon applying the staining conditions were monitored. Therefore, the phages MS2 and T7 were either incubated for 10 min at 80 °C or further processed without any temperature treatment. Ten µL of the sample were then applied to the carbon side of a carbon-coated copper grid. Excessive water was blotted dry with a filter paper and washed two times with double-distilled water. After each washing step grids were again blotted dry onto a filter paper before negative staining with 2% uranyl acetate for 20 sec. The staining liquid was blotted onto a filter paper and the grids were air-dried as described previously^33^. Transmission electron microscopy was carried out using a Zeiss EM 912 with an integrated OMEGA filter in zero-loss mode. The acceleration voltage was set to 80 kV and images were recorded using a Tröndle 2 k × 2 k slow-scan CCD camera (Tröndle Restlichtverstärker Systeme, Moorenweis, Germany).

### Sample stability test

In order to substantiate our decision of omitting a fixative step for FCM measurements and to confirm a certain stability of the virus concentration over a short time range (few days), phage T4 and wastewater samples were measured with FCM at time 0, after 24 h and after 48 h. The samples were either kept in 4 °C or were fixed with 0.5% glutaraldehyde for 30 min in 4 °C followed by freezing in liquid nitrogen with adjacent storage at -80 °C, as suggested by Brussaard (2004). At each time point, samples were prepared for FCM as described above with two different staining procedures (30 °C and 80 °C). Additionally, a fixed T4 phage sample was prepared for NTA measurements in the same way in order to test the usability of glutaraldehyde fixation. For phage T4, measurements of the 4 °C, unfixed samples were mostly slightly higher compared to the fixed samples (Fig. S6a,b). Comparing the initial quantification with the results after 48 h, the decrease in counted particles was minor. For the wastewater samples, viral numbers of the unfixed samples were marginally lower, however, a general decline in particle numbers over time could be observed (Fig. S6c,d). This decline was in all cases less than one order of magnitude. As both, fixed and unfixed samples declined only to a small extent and no trend of a stronger decrease of viral particles in the unfixed samples could be observed, omitting the fixation with glutaraldehyde and liquid nitrogen is not supposed to have a wide influence on the enumeration within 48 h.

### Statistical analysis

Statistical analysis was carried out in R (v3.4.3) and RStudio (v1.1.383). Data were log transformed and analysis of variance (ANOVA) was conducted. Normal distribution of data was confirmed by density plots and quantile–quantile plots; homogeneity of variances was confirmed with Levene's test. Afterwards, multiple pairwise comparisons were calculated with a *post-hoc* Tukey honest significant differences test. In addition, similarities in viral isolate quantification methods were assessed using principal coordinate analysis.

## Results

### Measurements of viral isolates

**Figure 1:**
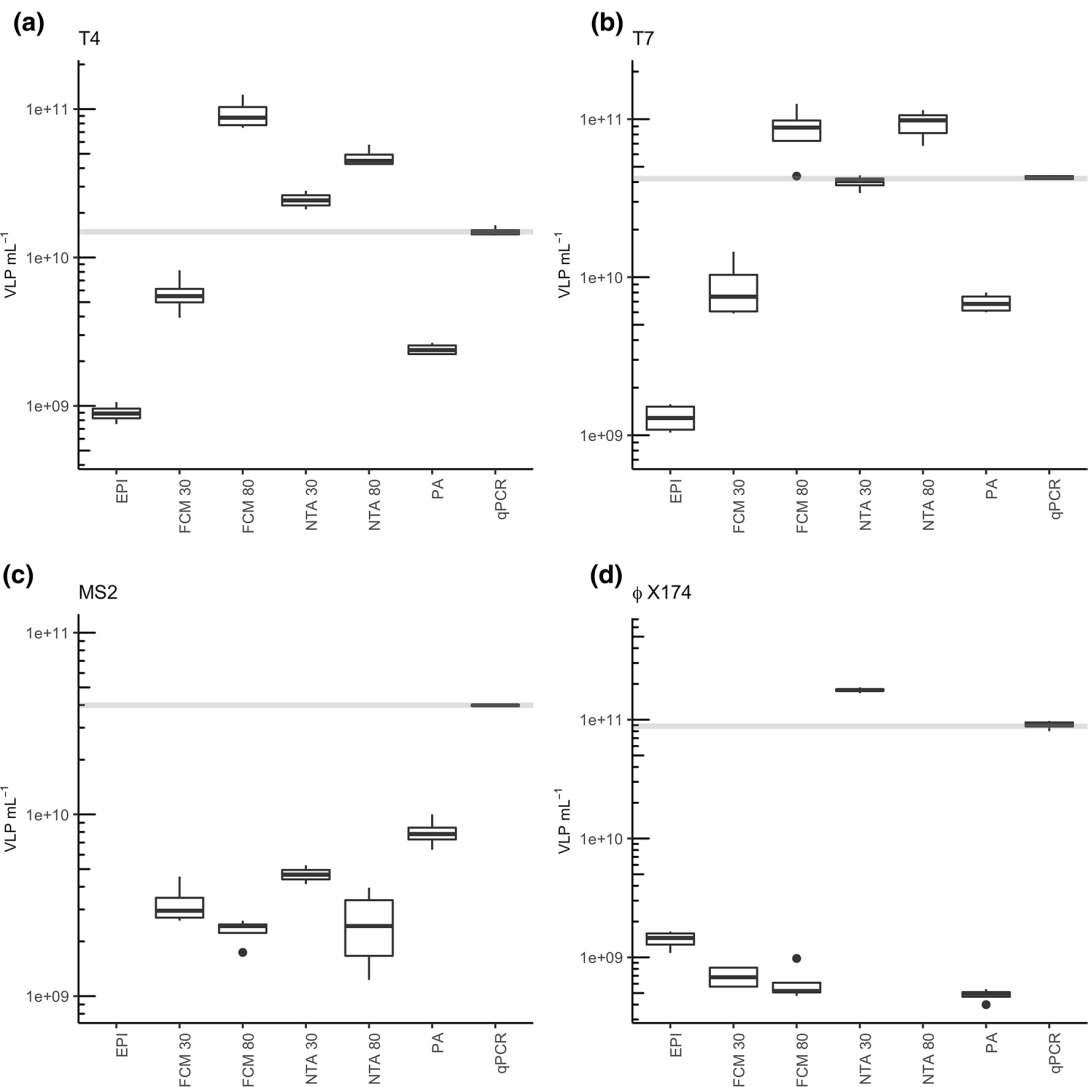
Enumeration of clonal viral isolates. Concentrations of (**a**) phage T4, (**b**) phage T7, (**c**) phage MS2 and (**d**) phage ϕ X174 in VLP mL^−1^ using different quantification techniques. Grey line represents median concentration measured by qPCR.

For the phage T4, qPCR gave a concentration of 1.52 × 10^10^ gene copies mL^–1^ (s = 1.02 × 10^8^, n = 8, R^2^ = 0.997; coefficient of determination) (Fig. 1a). However, EPI measurements gave a significantly lower result (p < 0.001). Likewise, concentrations measured with FCM in combination with staining at 30 °C (FCM30) and PA were lower. In both 80 °C staining treatments for FCM and NTA (FCM80 and NTA80), significantly higher particle concentrations were detected compared to qPCR (p < 0.001). For NTA measurements following a 30 °C staining procedure (NTA30), no significant difference was observed, despite a slightly elevated particle concentration.

A similar pattern appeared for the phage T7 (Fig. 1c). While qPCR measured 4.24 × 10^10^ gene copies mL^–1^ (s = 2.91 × 10^9^, n = 8, R^2^ = 0.988), EPI, FCM30, and PA gave significantly lower virus concentrations (p < 0.001). Here as well, staining at 80 °C significantly increased concentrations in both NTA and FCM (p < 0.001). The difference between the NTA30 and the qPCR result was negligible.

For phage MS2, qPCR quantification revealed 4.01 × 10^10 ^gene copies mL^–1^ (s = 1.26 × 10^9^, n = 8, R^2^ = 0.986) (Fig. 1e). Independent of the staining procedure, FCM significantly underestimated the particle concentrations (p < 0.001). Meanwhile, NTA30 measurements showed high imprecision with a general underestimation of particle numbers. With EPI, no concentration could be ascertained, as no particles were visible under the microscope at a magnification of 1000. In contrast to the other viral isolates, PA and NTA80 gave concentrations more similar to qPCR, but differences were significant (p < 0.001). Especially for NTA80, most of the counted particles were larger than 100 nm, which exceeds the actual MS2 diameter (Fig. S5).

According to qPCR, phage ϕX174 stock had a concentration of 8.69 × 10^10^ gene copies mL^–1^ (s = 9.45 × 10^9^, n = 8, R^2^ = 0.999) (Fig. 1g). For all other measurements, ANOVA tests showed significant differences (p < 0.001). For NTA30 and NTA measurement without fluorescent staining of the samples, significantly higher concentrations were obtained (p < 0.001). 80 °C staining of phage ϕX174 did not result in any countable particles during NTA measurements with all tracked particles showing a size of more than 500 nm (Fig. S5), which indicates a strong aggregate formation due to this high staining temperature.

Expectedly, PA consistently underestimated viral concentrations compared to qPCR, as with the latter method not only infectious but also non-infectious particles are quantified at the same time. Although broadly used in former studies for the enumeration of environmental (seawater) samples, countings with EPI returned rather low concentrations. The generally rather low concentrations of the small phages MS2 and ϕX174 measured with unspecific methods (EPI, FCM and NTA) compared to qPCR or PA results clearly show the requirements of these unspecific quantification techniques in terms of particle sizes. With phages T4 and T7, both with a diameter of more than 50 nm, more reasonable results were obtained using unspecific methods.

**Figure 2:**
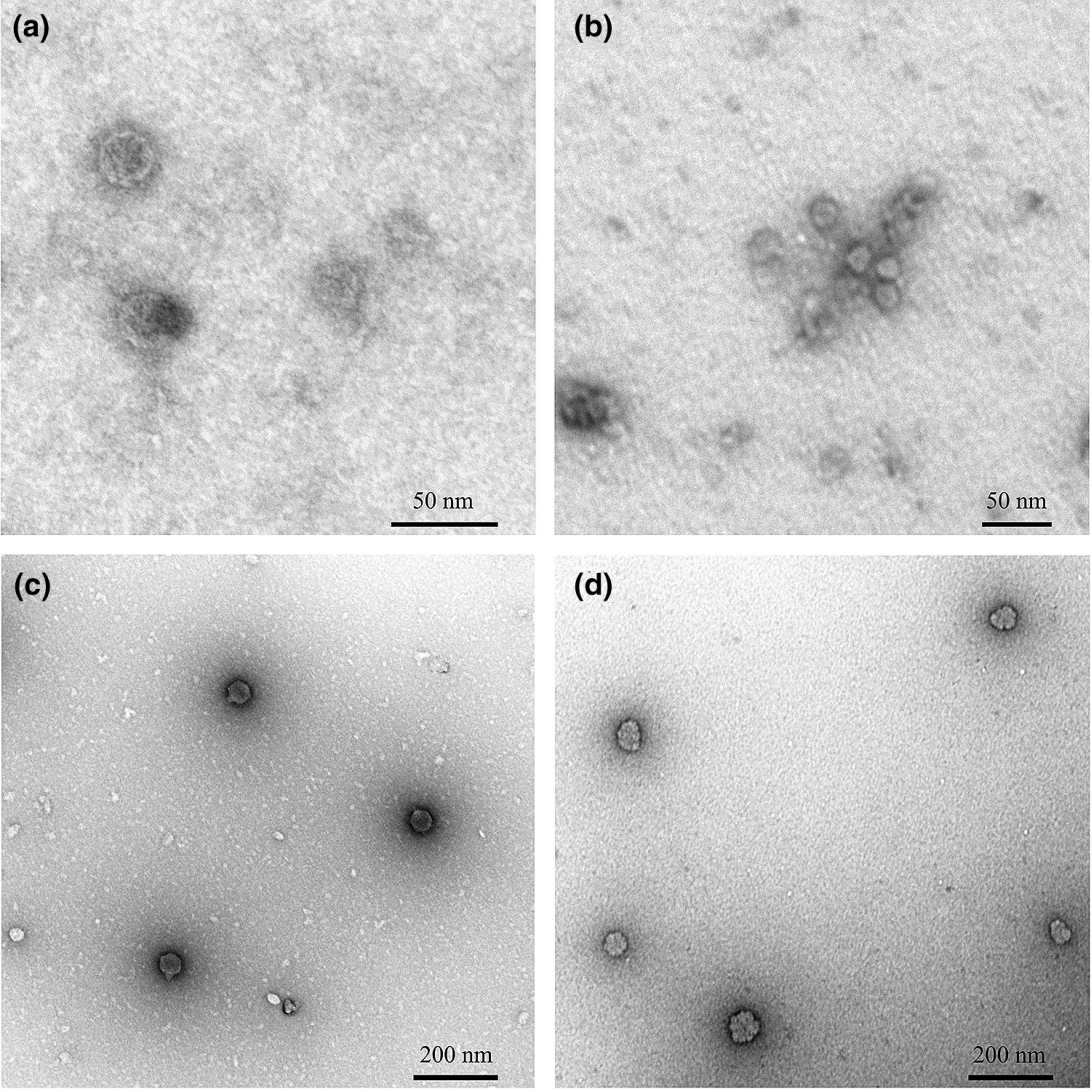
Morphology of viral isolates using transmission electron microscopy (TEM). (**a**) phage MS2 with a scale bar of 50 nm, untreated; (**b**) phage MS2 with a scale bar of 50 nm, incubated at 80 °C for 10 min; (**c**) phage T7 with a scale bar of 200 nm, untreated; and (**d**) phage T7 with a scale bar of 200 nm, incubated at 80 °C for 10 min.

**Figure 3:**
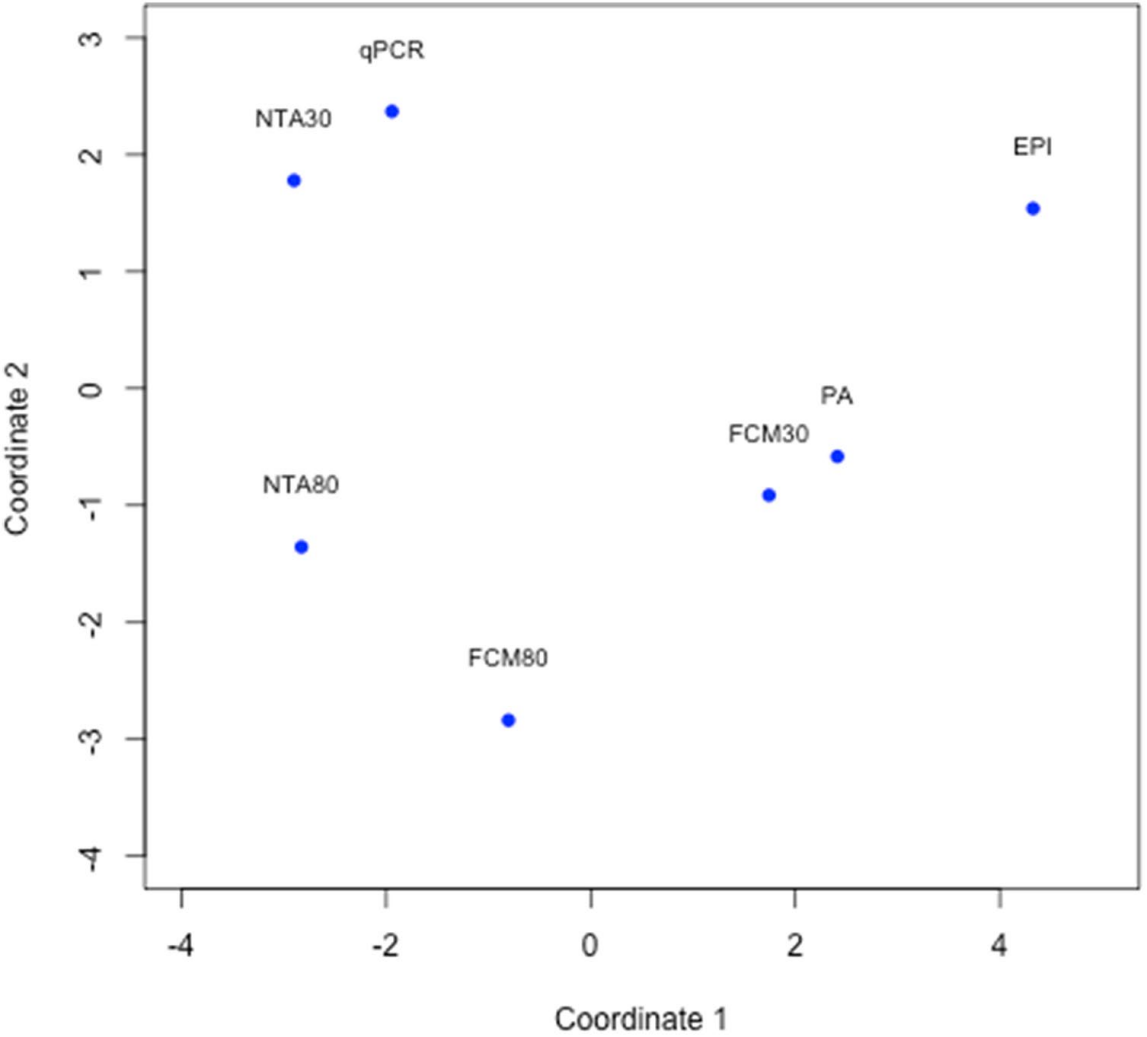
Principal coordinate analysis (PCoA) of all viral isolates indicating a correlation between the quantification methods qPCR and NTA30.

The highest particle concentrations for phage T4 and T7 were quantified using the 80 °C staining procedure with both methods, NTA and FCM. However, treatment at 80 °C also resulted in an altered size distribution of the viral particles that did not reflect the actual particle sizes. This became especially apparent for the very small phages MS2 and ϕX174, as NTA80 was not able to record particles with an actual size of 30 nm (Fig. S5f). Indeed, for MS2 all tracked particles showed a size of more than 100 nm with most of the particles even greater than 500 nm. For phage ϕX174 no particles with a size of 30 nm could be counted at all, as only particles far beyond this size were tracked (data not shown). Hence, also measurements of stained samples at 80 °C with FCM have to be handled with care. The effect of the high temperature staining has been further evaluated using TEM recordings of phages MS2 and T7, untreated or incubated at 80 °C for 10 min (Fig. 2). Although singular MS2 particles appeared smaller when incubated at 80 °C (29.20 ± 7.18 nm) compared to untreated phage particles (34.78 ± 8.23 nm), they did show a tendency to form aggregates (Fig. 2a,b). For phage T7, this aggregation formation was less apparent, but particles lost their distinct icosahedral shape upon the incubation at 80 °C (Fig. 2c,d). Although this 80 °C staining procedure is widely used for the quantification of viral particles^14,34^, it may lead to an increased aggregation of the viral particles and thus to imprecise measurements. A gentler staining treatment at 30 °C led to underestimations of the viral particle concentration using FCM for quantification. Indeed, only NTA in combination with 30 °C staining resulted for the phages T4 and T7 in concentrations similar to qPCR results, which was also verified by ANOVA tests, that showed no significant deviation. This congruency could also be confirmed by principal coordinate analysis (PCoA) analysis, in which a clustering of NTA30 and qPCR was observable (Fig. 3).

### Measurements of environmental samples

**Figure 4:**
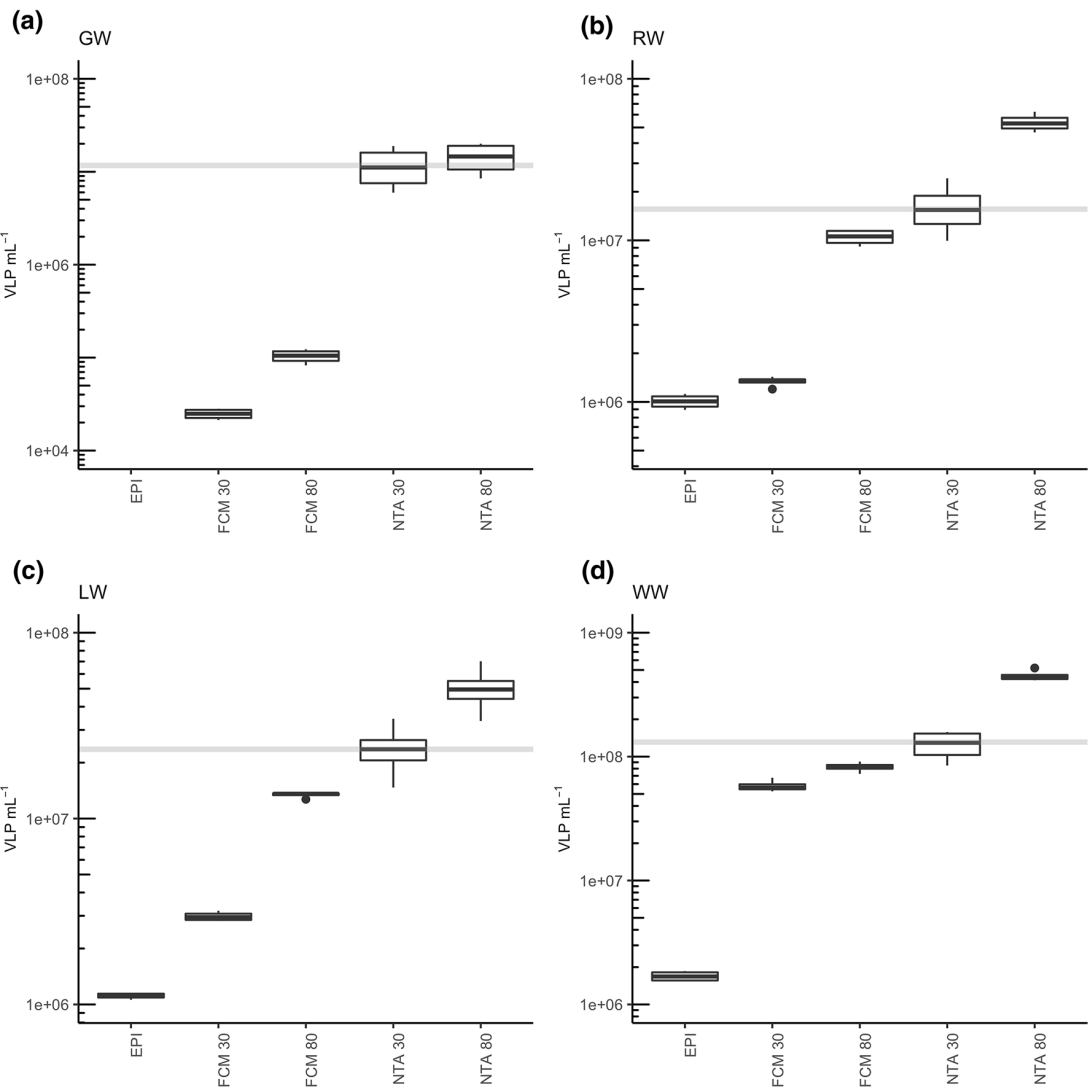
Enumeration of viral particles in environmental water samples. (**a**) Groundwater (GW); (**b**) lake water (LW); (**c**) river water (RW); (**d**) wastewater (WW). Grey lines indicate the median particle concentration measured using NTA30.

For groundwater, NTA30, NTA80, and NTA unstained achieved concentrations of approximately 10^7^ VLP mL^–1^ (Fig. 4a). With EPI, no concentration could be ascertained, as the particles showed a fluorescence signal too weak to be visible. Both FCM30 and FCM80 gave lower particle numbers of around 10^5^ VLP mL^–1^.

The river water sample was quantified with NTA30 to a concentration of 10^7^ VLP mL^–1^ (Fig. 4b). A similar result was obtained with FCM80. Both NTA80 and NTA unstained gave ten-fold higher concentrations, whereas EPI and FCM30 showed ten-fold lower concentrations around 10^6^ VLP mL^–1^.

For the lake water sample, viral concentrations were similar to the river water (Fig. 4c). The lowest viral concentration (10^6^ VLP mL^–1^) was determined by EPI, whereas NTA unstained gave the highest virus concentration (10^8^ VLP mL^–1^).

The virus concentration for the wastewater with NTA30 was 10^8^ VLP mL^–1^ (Fig. 4d). Higher concentrations were indicated by NTA80 and NTA unstained. The lowest concentration of 10^6^ VLP mL^–1^ was obtained by EPI. Neither FCM30 nor FCM80 showed considerable differences from NTA30 (10^8^ VLP mL^–1^).

The complex and heterogeneous nature of environmental water samples, together with a background rather high in abiotic particles, presents challenges to accurate virus quantification. Besides, targeted methods are not applicable when the viral community in its entirety has to be enumerated. It is therefore difficult to come to the decision which method is most reliable for the quantification of environmental water samples. None of the tested methods was able to provide absolute values, as the exact concentration of the viral particles in these samples is virtually impossible to determine. All non-targeted methods (FCM, EPI and NTA) only quantify the nucleic acid stains and not the particles themselves. Therefore, all methods have the potential for inaccuracies, e.g. through the erroneous quantification of small bacteria or extracellular vesicles as described in Sawaya et al*.*^35^. The previous analysis of different viral stocks however indicated, that NTA30 returns concentrations most similar to qPCR results. Quantifications of the environmental samples with NTA80 showed the tendency to overestimate the viral concentrations, whereas FCM30 as well as EPI lead to underestimations. Both FCM80 and NTA30 are more similar in their results, with slightly lower viral concentrations quantified with FCM80. From the results it is however impossible to identify the best method for the quantification of viral particles. When a defined viral particle with known bacterial or eukaryotic host or known viral genome has to be enumerated, plaque or cultivation-based assays of qPCR are the methods of choice. For all other applications where unknown viral mixtures shall be quantified, NTA30 or FCM80 seem to be the best options. The results with FCM80 however have to be handled with care, as with this harsh incubation conditions at very high temperatures, particles tend to form aggregates which impair precise measurements.

A further element of uncertainty is the lack of ability for the enumeration of very small viral particles as it could be seen with the phage stocks MS2 and ϕX174. From the results of the stock quantifications it is very questionable, to which extent small viral particles are captured with NTA or FCM. Both methods underestimated the actual particle concentrations independent of the applied staining procedure. These laser-based methods rely on the incorporation of sufficient dye molecules into the genome in order to return visible signals, which is often not enough in such small genomes. Similar observations have been made with viruses less than 40 nm in diameter using SYBR green I^36^. SYBR gold is widely used for the staining of environmental samples in order to enumerate viral particles in their entirety^14^. It is however questionable to what extent this dye is suitable for staining ssDNA or RNA particles (such as ϕX174 or MS2), as the accuracy of NTA or FCM was both not sufficient with these viruses. For the assessment of these virus types, more specific nucleic acid dyes should be evaluated in further studies.

A last aspect important for optimal results are the requirements each method has for the minimal particle concentration. For groundwater, both FCM30 and FCM80 gave concentrations between 10^4^ and 10^5^ VLP mL^–1^, which is in the range of previously ascertained groundwater samples obtained FCM in combination with SYBR green I staining^37^. Paradoxically, for the groundwater samples measured in this study, all NTA measurements (independent of staining procedure) gave concentrations of around 10^7^ VLP mL^–1^. This discrepancy between FCM and NTA could be because the optimal concentration required by NTA lies between 10^7^ and 10^8^ mL^–1^ (Table 1). For the calculation of particle concentration, the volume in the measuring chamber is extrapolated. In case of very low particle amounts, a high random error appears, especially when no syringe pump is used. Additionally, the signal-to-noise-ratio turns to be unfavorable when measuring at such low numbers. Consequently, NTA is incapable to accurately quantify a sample if its concentration is much less than the minimal required value. Thus, an application of NTA to oligotrophic groundwater is unfeasible without prior enrichment. However, varying optimal concentrations are required for different instruments. Optimal concentrations for FCM quantification vary with the complexity of samples. Clonal isolates are generally featured with lower background signals, thus virus concentrations around 10^6^ to 10^7^ particles mL^–1^ are optimal. For the highly variable environmental samples, lower virus concentrations (10^4^ VLP mL^–1^) were needed, as the background noise is then diluted, too. For NTA, these differences could be observed as well, although not as pronounced. Viral isolates need a final concentration of 10^8^ VLP mL^–1^ for medium-sized viral particles (e.g., phages T4 or T7) or 10^9^ VLP mL^–1^ for smaller particles (e.g., phages MS2 or ϕX174). Environmental samples need a minimal concentration of 10^7^ VLP mL^–1^ for optimal results. For PA, samples were diluted to result in a countable plaque formation in the range of 20–200 plaque forming units (PFU) per plate. However, this number may be different between phage isolates as the plaque diameter also varies individually. Lower concentrations on the other hand may lead to pronounced variations between replicates. qPCR is theoretically capable to detect one gene copy per reaction volume. Thus, its sensitivity is more restricted to the efficiency of DNA or RNA extraction and cDNA synthesis, than to the limitations of the quantification method. For EPI, concentrations of 10^7^ particles mL^–1^ are generally necessary in order to being countable. A total magnification of 1000 is however not sufficient for a reasonable enumeration of very small viruses (e.g., phages MS2 or ϕX174), although the amount of added dye was doubled for the EPI sample preparation compared to FCM or NTA, indicating that this increment does not improve particle visibility.

### Measurements of the mixed environmental samples spiked with phage T4

**Figure 5:**
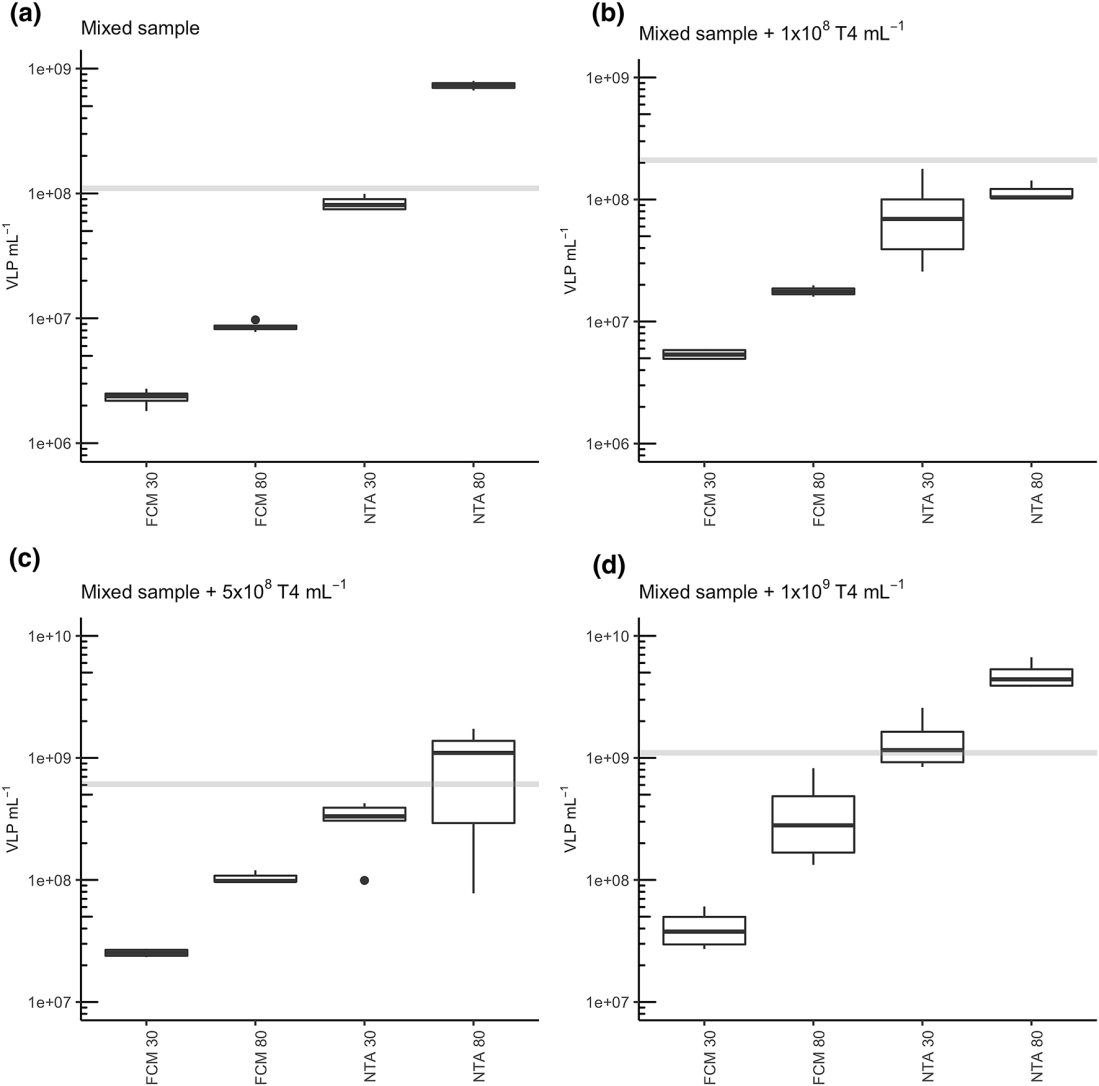
Enumeration of viral particles in mixed water samples spiked with different phage T4 concentrations. (**a**) Mixed water; (**b**) mixed water spiked with 1 × 10^8^ T4 mL^−1^; (**c**) mixed water spiked with 5 × 10^8^ T4 mL^−1^; (**d**) mixed water spiked with 1 × 10^9^ T4 mL^−1^. Grey lines indicate the expected total virus concentration for each sample according to the initial enumeration of the environmental sample with NTA30 in addition to the artificially added phage T4.

In a further attempt to compare and test NTA and FCM, an environmental sample has been spiked with different concentrations of phage T4. The initial virus particle concentration of the environmental samples has been estimated using NTA30. The concentration of the mixed environmental sample (a mixture of river and waste water) has then been adjusted to reach an approximate concentration of 1 × 10^8^ VLP mL^−1^. To this sample, different concentrations of phage T4 (quantified by qPCR) have been added (1 × 10^8^, 5 × 10^8^ and 1 × 10^9^ VLP mL^−1^). These samples were quantified with NTA and FCM in combination with the two different staining procedures (30 °C and 80 °C). The mixed water sample without artificially added phage particles has been quantified using NTA30 to 1.1 × 10^8^ VLP mL^−1^ (Fig. 5a). Both, FCM30 and FCM80 underestimated the viral particle concentration by 47.4- and 12.8-fold, respectively. NTA80, however, overestimated the viral concentration by 6.7-fold, whereas NTA30 underestimated the expected viral concentration with 1.3-fold only slightly. A similar pattern could be observed for the mixed sample with 1 × 10^8^ T4 particles mL^−1^ added (Fig. 5b). FCM measurements underestimated the virus concentration by 38.9- (FCM30) and 11.8-fold (FCM80). NTA80 quantified 5.9-fold more viral particles and NTA30 resulted with a 2.6-fold lower concentration to the closet titer estimation. Figure 5c represents the measurements of the mixed sample spiked with additional 5 × 10^8^ T4 particles mL^−1^. High deviations from the expected concentration of 6.1 × 10^8^ VLP mL^−1^ could again be observed for both FCM measurements. NTA30 slightly underestimated the concentration (2.0-fold) whereas NTA80 overestimated the particle concentration by 1.6-fold. With a starting concentration of 1 × 10^9^ VLP mL^−1^, only NTA30 resulted in the closest estimation of the particle concentration with a slight overestimation of 1.3-fold (Fig. 5d).

With this experiment, previous findings could be confirmed. Both, FCM 80 (to a lower extent) and FCM30 (to a higher extent) underestimated the viral particle concentration, whereas NTA80 led to overestimations. Assuming an optimal particle concentration, NTA together with 1 h staining at 30 °C could be reassured as a reliable quantification technique for environmental samples on the condition, that a minimum particle concentration of 10^7^ VLP mL^–1^ is reached.

## Conclusion

This study compared five different quantification methods on four viral strains (with different genome types) as well as on four environmental water samples. A differentiation has to be made between rather pure viral isolates and complex environmental samples, that harbor the potential for high background noise. Whereas for the viral isolates, targeted methods (e.g. PA, qPCR or the polony method^11^) return accurate numbers, environmental samples still lack an universally applicable and accurate quantification method. This comes also along with the need for optimizing and adapting protocols for different sample types (e.g. samples with high or low expected virus concentrations, or with high background noise). One conclusion that can be drawn from the measurements of the environmental sample is the insufficient suitability of applying an 80 °C staining temperature due to the increased formation of viral aggregates. NTA in combination with a staining procedure at 30 °C was however the only method that resulted in values with a high accordance to qPCR results. Other methods or staining protocols such as EPI, NTA80 or FCM30 enumerated either too many or too less particles. In contrast to FCM, no glutaraldehyde fixed samples can be measured with NTA, implying an immediate (within few days) sample processing. It may be worthwhile to study different sample preservation methods for their suitability for NTA measurements to enhance the applicability of NTA. A further drawback of NTA compared to FCM is the time needed for sample measurements, as here, no high throughput is possible. NTA allows on the other hand a visual control of what is counted and with that also allows for drawing conclusions on the reliability of the results, as it could be seen for the small viruses MS2 and ϕX174 with their unreasonable size distribution (Fig. S5f) at higher staining temperature. Besides the necessity of testing different sample preservation methods, also different nucleic acid dyes or protein dyes may be evaluated, or even the application of both. With that, the specificity of fluorescence based methods (EPI, FCM or NTA) may be enhanced and the visibility of viral particles may be improved. The challenge very small viral particles (less than 50 nm) impose on the quantification still remains. From the methods tested in this study it is questionable to what extent small viral particles are captured. Here, none of the tested methods offers a solution. Each method has its requirements for minimal particle concentrations and particle sizes which have to be met to ensure reliable results. The ineffectiveness in quantifying especially small viral particles with FCM, NTA or EPI, resulting in underestimations of viral titer, has an impact on estimations of global viral numbers extrapolated according to measurements obtained previously by similar methods.

The global viral population in seawater has been estimated as 4.1 × 10^30^ VLP^10^. This value was based on epifluorescence microscopic counting using Yo-Pro1 dye. Clasen et al*.* quantified the viral population in freshwater and obtained a concentration of 9.5 × 10^9^ VLP L^–1^ using the same method and dye^38^. Assuming a global freshwater volume of 9.0 × 10^16^ L (including lakes and rivers), the viral freshwater population can thus be calculated as 8.6 × 10^26^ VLP^39^. However, in the present study we observed a general underestimation of viral numbers in a given sample, especially by EPI, as well as difficulties in quantifying very small particles. One important consideration regarding these significant underestimations is the chemical properties of the fluorescent dyes applied in the aforementioned and the present study. Yo-Pro1, SYBR gold, and SYBR green are dyes that selectively stain dsDNA. RNA viruses or ssDNA viruses are therefore stained only weakly or not at all. As Steward and colleagues showed earlier, only one-third of the viruses in seawater are dsDNA viruses, whereas up to 63% are RNA viruses^40^. With this high proportion of particles that are beyond the scope of conventional methods, it is reasonable to assume that more than half of the particles are not captured using EPI in combination with these dyes. Previous speculated values of the total viruses in a given environment, which are based on epifluorescence counting, have to be reconsidered as they likely lead to underestimations of global virus abundance.

## Supplementary information

Supplementary Information

## Data Availability

The collected data are available through the following link: https://osf.io/xtg2h/ (https://doi.org/10.17605/OSF.IO/XTG2H).
