# Supplementary material for: A rigorous assessment and comparison of enumeration methods for environmental viruses
Source: Sci Rep. 2020 Oct 29;10:18625. doi: 10.1038/s41598-020-75490-y (PMC7596560; doi:10.1038/s41598-020-75490-y)
Supplement: Supplementary file 1 — Supplementary Information [file 41598_2020_75490_MOESM1_ESM.docx]

**A rigorous assessment and comparison of enumeration methods for environmental viruses**

Judith Feichtmayer^1,2,3^, Carolin Pickl^5^, Christian Griebler^3,4^, Andreas Klingl^5^, Rainer Kurmayer^6^ , Li Deng^1,2^*

^1^Institute of Virology, Helmholtz Centre Munich, Germany

^2^Institute of Virology, Technical University Munich, Germany

^3^Institute of Groundwater Ecology, Helmholtz Centre Munich, Germany

^4^Department for Limnology and Bio-Oceanography, University of Vienna, Austria

^5^Plant Development and Electron Microscopy, Department Biology I, Biocenter, Ludwig-Maximilians-University Munich, Germany

^6^Research Department for Limnology, University of Innsbruck, Austria

**Running title:** Environmental viral enumeration

***** **Corresponding author:**

Email: [li.deng@helmholtz-muenchen.de](mailto:li.deng@helmholtz-muenchen.de)

# Supporting Information

## qPCR set-up optimization

As qPCR has been used as golden standard for this manuscript, special attention has been put on the set-up and optimization of the optimal conditions. Initially, a range (0.05 µM – 1 µM) of different concentrations of the forward and reverse primer were tested in order to find the optimal primer concentration for each phage type. The following concentrations for both, forward and reverse primer, have been found to be optimal under the applied thermal conditions: T4: 0.5 µM T4F and T4R; T7: 0.8 µM T7_4453F and T7_5008R; φX174: 0.6 µM φX174F and φX174R; MS2: 0.3 µM MS2_2717F and MS2_3031R.

## Synthesis of DNA standards

The applied standards were purchased from IDT (Coralville, Iowa). These gblock gene fragments were individually synthesized for each phage type for the specific region that is amplified by the applied forward and reverse primer plus additional ten nucleotides before and after the forward or reverse primer, respectively. The standard DNA fragments comprised of the following sequences (the primer sequences are underlined; 5’ – 3’). The correct size has been verified using capillary electrophoresis and the correct sequence has been identified by mass spectrometry, both tests performed by the company:

For T4: AATTACGCTAAAGCGAAAGAAGTCGGTGAATATCCAACACTAGGTTCTAACTGGACTGCGGAAATTTCTTCATCTTCCTCTGGTTTAGCTGCAGTAATAACTCTTGGAAAAATTATTACTGATTCTGGTATTTTATTAGCTGAAATTGAAAATGCTGAAGCTGCTATGACAGCGGTTGACTTTC

For T7:

CGTGTTTACGCTGTGTCAATGTTCAACCCGCAAGGTAACGATATGACCAAAGGACTGCTTACGCTGGCGAAAGGTAAACCAATCGGTAAGGAAGGTTACTACTGGCTGAAAATCCACGGTGCAAACTGTGCGGGTGTCGATAAGGTTCCGTTCCCTGAGCGCATCAAGTTCATTGAGGAAAACCACGAGAACATCATGGCTTGCGCTAAGTCTCCACTGGAGAACACTTGGTGGGCTGAGCAAGATTCTCCGTTCTGCTTCCTTGCGTTCTGCTTTGAGTACGCTGGGGTACAGCACCACGGCCTGAGCTATAACTGCTCCCTTCCGCTGGCGTTTGACGGGTCTTGCTCTGGCATCCAGCACTTCTCCGCGATGCTCCGAGATGAGGTAGGTGGTCGCGCGGTTAACTTGCTTCCTAGTGAAACCGTTCAGGACATCTACGGGATTGTTGCTAAGAAAGTCAACGAGATTCTACAAGCAGACGCAATCAATGGGACCGATAACGAAGTAGTTACCGTGACCGATGAGAACACTGGTGAAATCTCTGAGAAAGTCAAGCTGGGCACTAAGGCACTG

For MS2:

CCATGATATTCTGGGCAATAGTCAAAGCGACCCAAATCCATTTTGGTAACGCCGGAACCATAGGCATCTACGGGGACGATATTATATGTCCCAGTGAGATTGCACCCCGTGTGCTAGAGGCACTTGCCTACTACGGTTTTAAACCGAATCTTCGTAAAACGTTCGTGTCCGGGCTCTTTCGCGAGAGCTGCGGCGCGCACTTTTACCGTGGTGTCGATGTCAAACCGTTTTACATCAAGAAACCTGTTGACAATCTCTTCGCCCTGATGCTGATATTAAATCGGCTACGGGGTTGGGGAGTTGTCGGAGGTATGTCAGATCCACGCCTCTATAAG

For φX174:

CTGACGAGTAACAAAGTTTGGATTGCTACTGACCGCTCTCGTGCTCGTCGCTGCGTTGAGGCTTGCGTTTATGGTACGCTGGACTTTGTAGGATACCCTCGCTTTCCTGCTCCTGTTGAGTTTATTGCTGCCGTCATTGCTTA

## Calculation of DNA standard templates

The standards have been reconstituted according to the manufacturer’s information with 1× TE buffer (pH 7.5, AppliChem). The exact DNA concentration has been quantified using the Quant-iT PicoGreen DNA Kit (Thermo Fisher) according to the manufacturer’s protocol. As a standard, eight dilutions of the supplied lambda DNA were used in the range of 0 to 2.5 ng µL^-1^. Based on these this quantification results, the respective gene copy number per µL for each standard was calculated as followed:

S1 Table: Amplicon per µL calculation of standard DNA template.

| Phage | Size of amplicon (nt) | Da/Amp | ng / µL | Amp / µL |
| --- | --- | --- | --- | --- |
| T4 | 164 | 101350.0 | 12.00 | 7.13E+10 |
| T7 | 556 | 343593.3 | 8.32 | 1.46E+10 |
| MS2 | 315 | 194674.0 | 5.07 | 1.57E+10 |
| φX174 | 123 | 76038.2 | 4.33 | 3.43E+10 |

For each qPCR measurement, standard DNA templates have been serially diluted in ten-folds to range between the magnitudes E+01 to E+08.

The amplification efficiencies and the respective coefficients of determination for each phage are as followed:

S2 Table: Amplification efficiency and coefficient of determination.

| Phage | slope of standard curve | amplification efficiency | coefficient of determination (R^2^) |
| --- | --- | --- | --- |
| T4 | -3.578 | 90.3 % | 0.997 |
| T7 | -3.509 | 92.7 % | 0.987 |
| MS2 | -3.428 | 95.8 % | 0.986 |
| φX174 | -3.403 | 96.7 % | 0.999 |


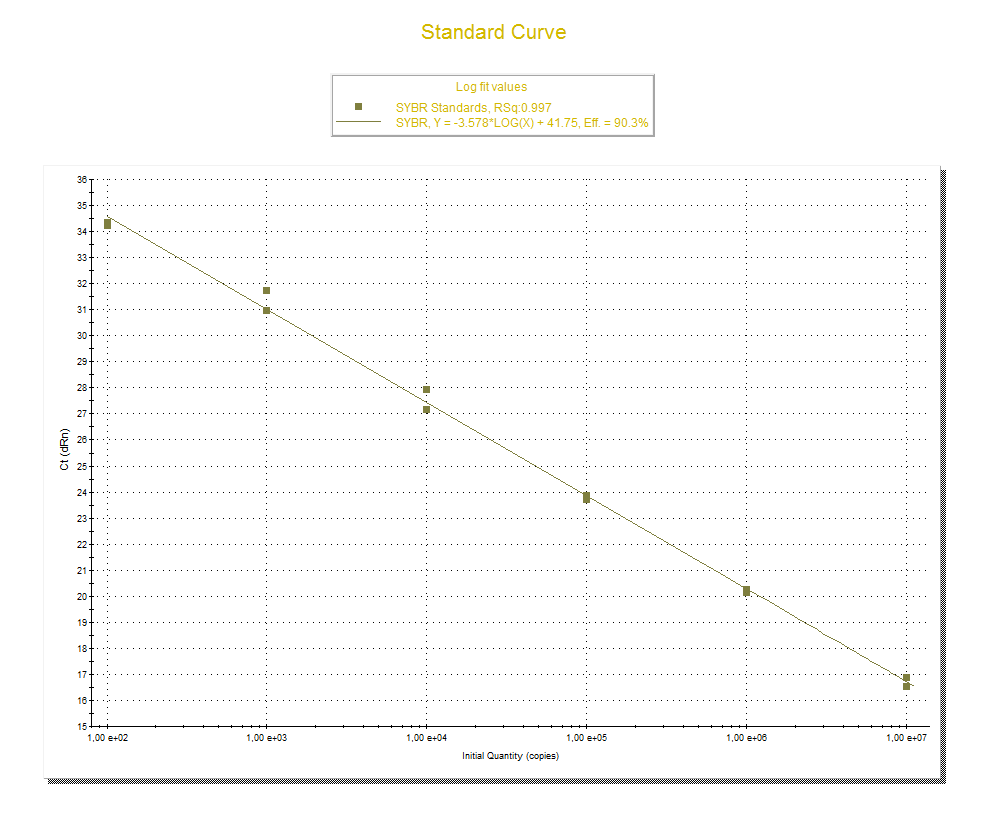


S1 Fig: Standard curve of phage T4 qPCR.


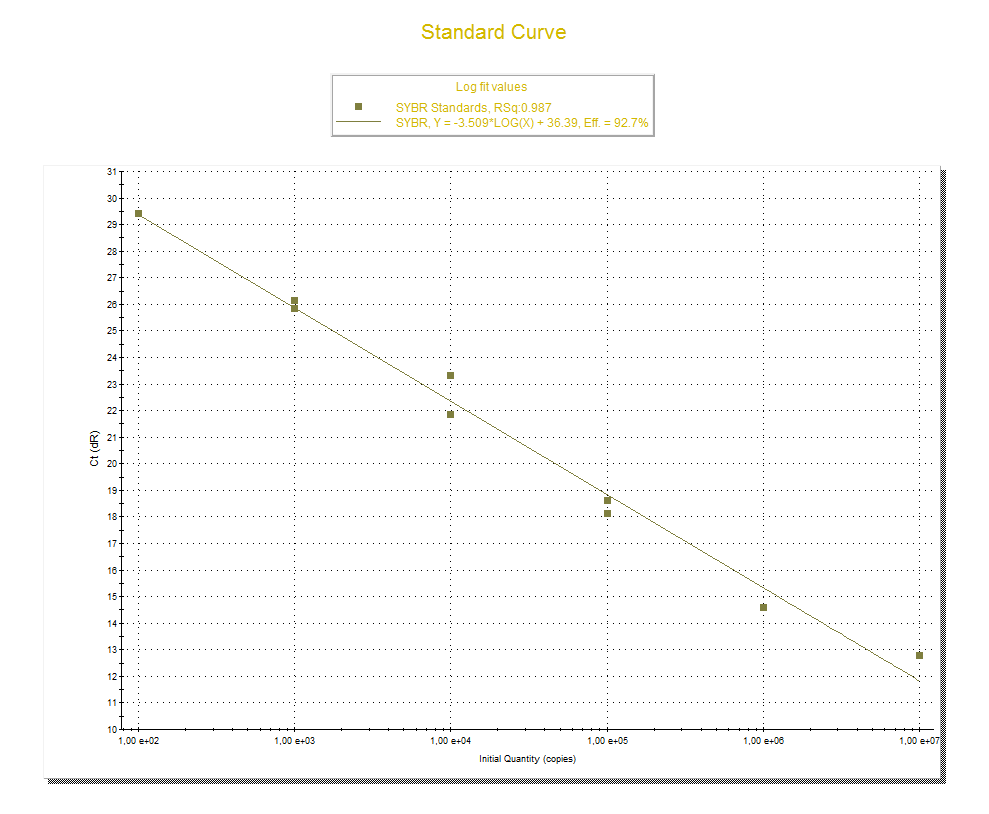


S2 Fig: Standard curve of phage T7 qPCR.


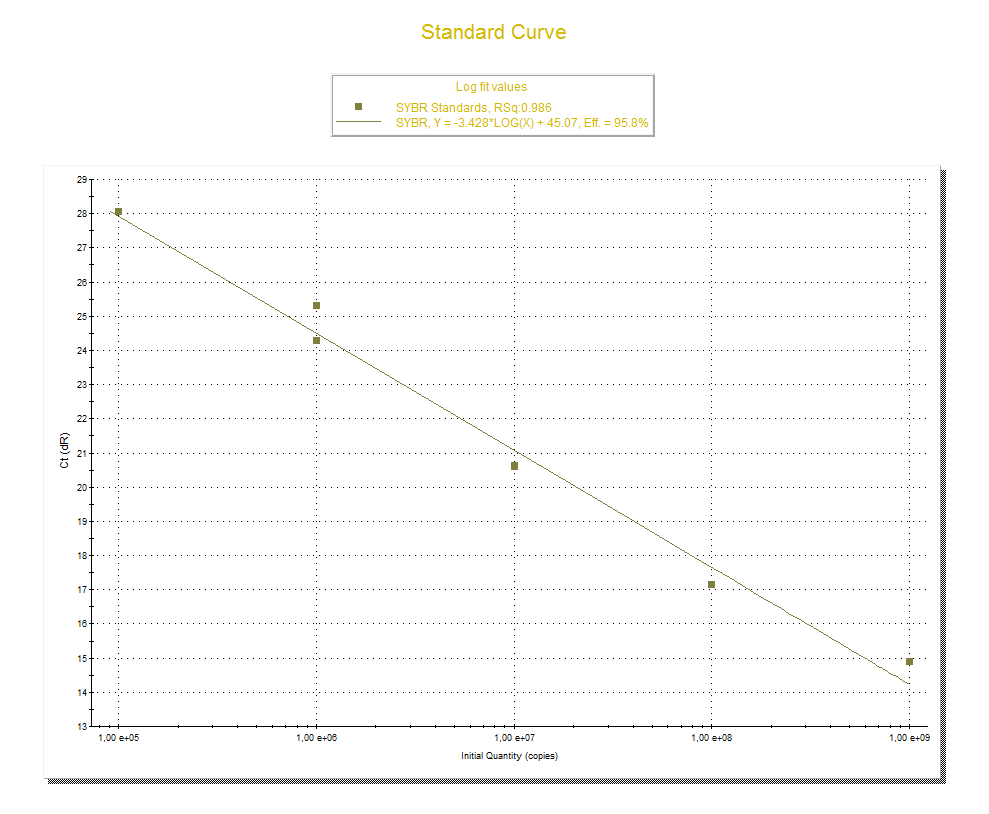


S3 Fig: Standard curve of phage MS2 qPCR.

 
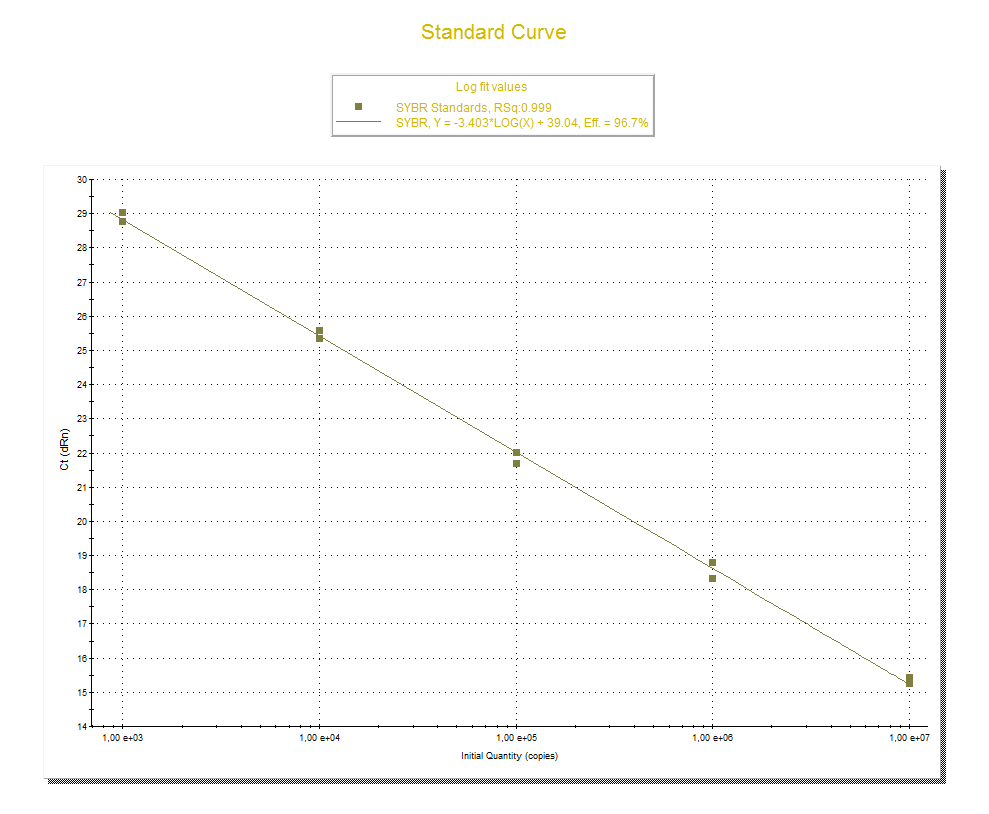


S4 Fig: Standard curve of phage φX174 qPCR.


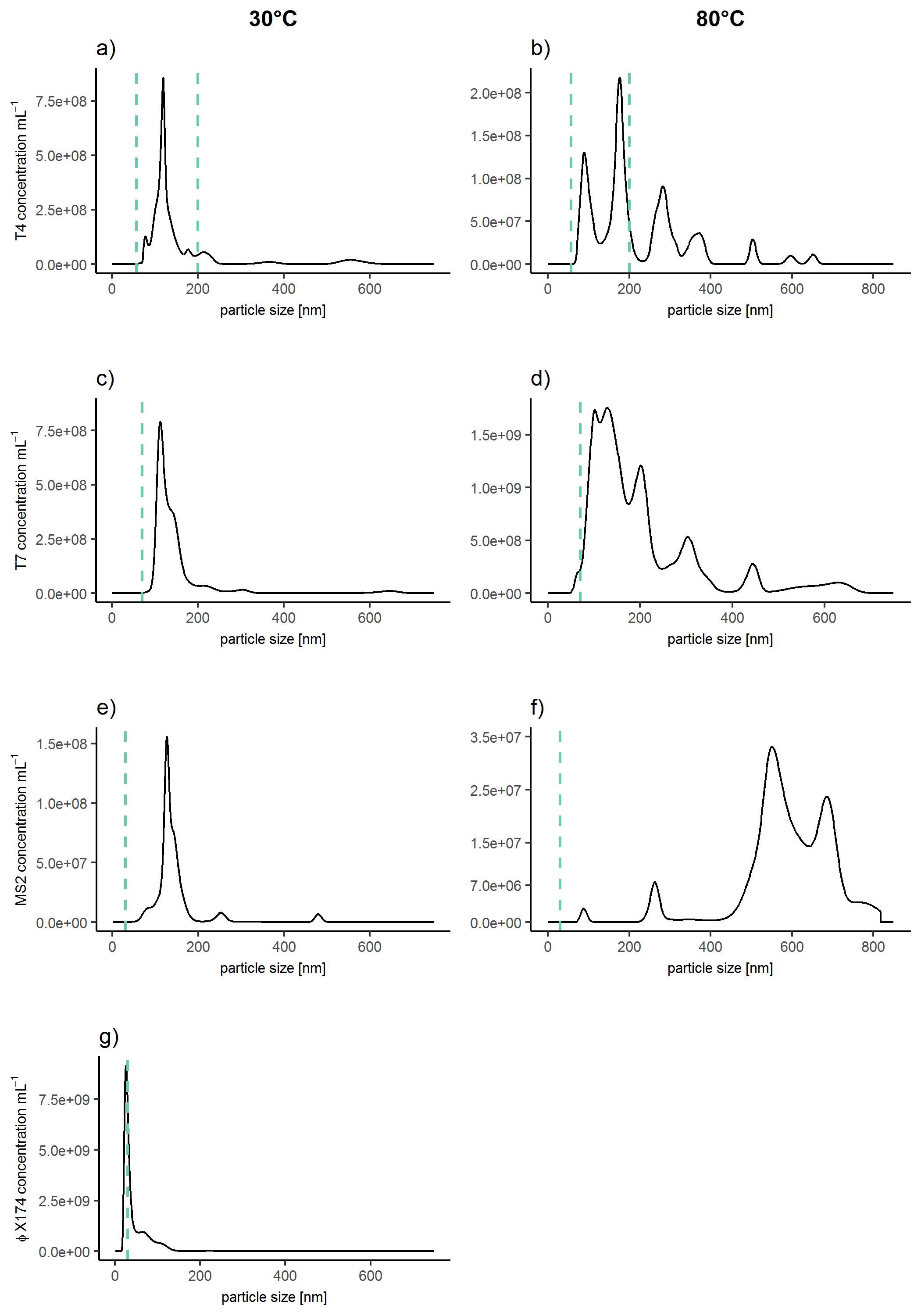


S5 Fig: Size distribution of viral isolates quantified with NTA method. (a), (c), (e) and (g): size distribution with 30°C staining of phages T4, T7, MS2 and φX174, respectively. (b), (d), (f) and (h): size distribution with 80°C staining of the respective phages. Dotted green line indicates the actual particle size of the respective virus isolate (T4: range of 57 – 200 nm, T7: 70 nm, MS2: 30 nm, φX174: 30 nm). Concentrations represent the average of three measurements of one sample preparation.


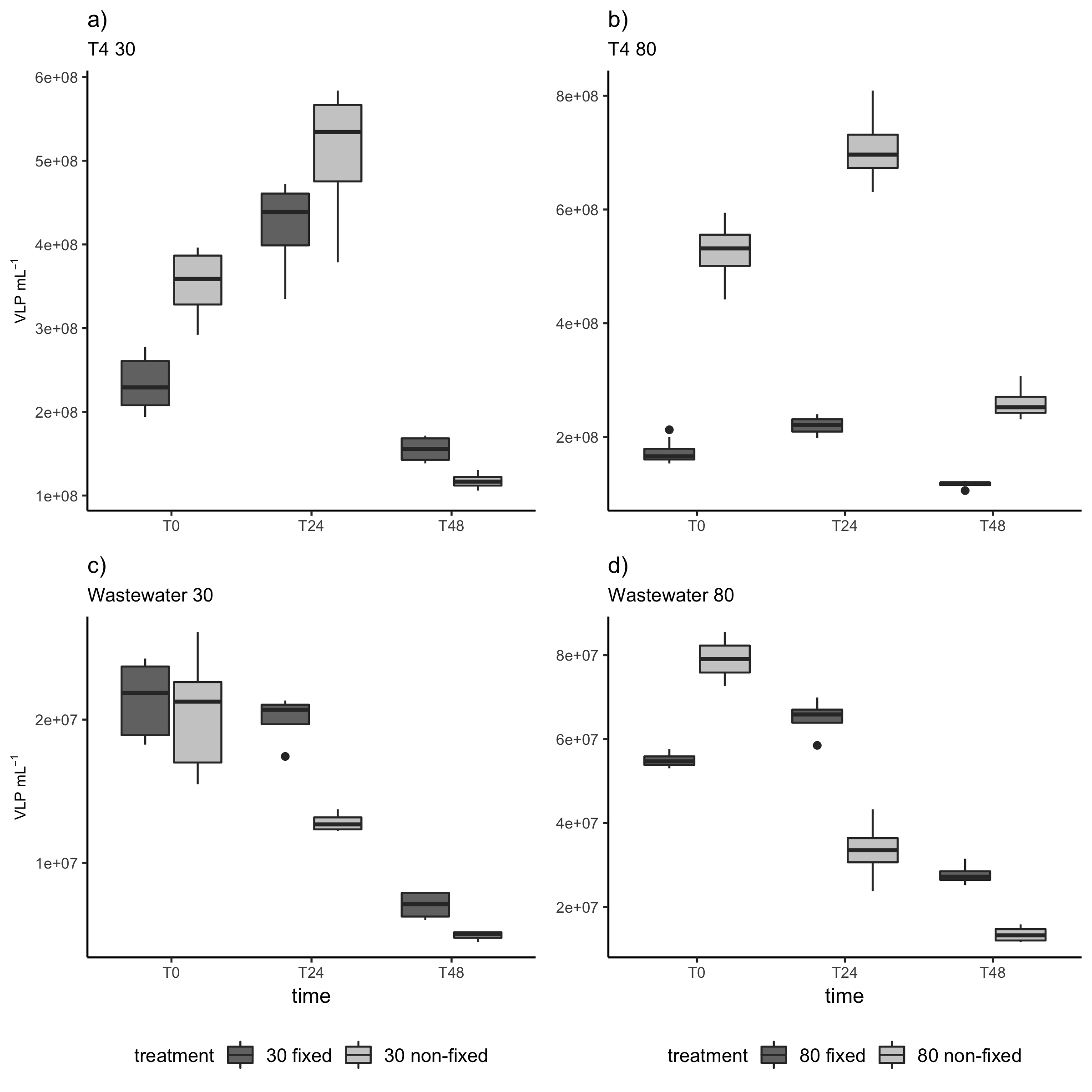


S6 Fig: Fixed (glutaraldehyde and liquid nitrogen, stored in -80°C) and unfixed (stored in 4°C) samples of phage T4 and wastewater, measured at time 0, after 24 h and 48 h. (a) Phage T4, stained at 30°C; (b) phage T4, stained at 80°C; (c) wastewater stained at 30°C; (d) wastewater stained at 80°C.
